# Extracts from Medical Clinic of Prof. Jas. T. Whittaker

**Published:** 1883-03

**Authors:** 


### SELECTIONS.

### Extracts from Medical Clinic of Prof. Jas. T. Whittaker.

Erysipelas never comes without a break somewhere in the skin, a solution of continuity. Formerly erysipelas was divided into idiopathic and traumatic varieties, the former supposed to originate of its own accord, whilst the latter was produced by some accident or surgical injury. Trousseau first showed that erysipelas is always traumatic. The disease shows itself on the skin, sometimes on the mucous membrane internally, but it always stands in connection with the outer world and is always produced by a solution of continuity. It often begins about the angles of the nose, mouth, or eye, from a slight abrasion frequently overlooked or perhaps healed over at the time. It is most frequent in this region where it is called erysipelas faciei. Erysipelas capitis, generally begins too in the face and then travels up. Sometimes the break is healed up and it might be thought that it entered the unbroken skin. This is never the case; there is always a delicate scar sufficient to allow of the permeation of the germs from which the disease develops. It does not necessarily complicate the healing process of a wound, though it sometimes leads to gangrenous destruction. Busch reports interesting cases of malignant tumors, especially lupus healed rapidly by an erysipelas. The new infection substitutes and supplants the old inflammation, or disease process.

Fugacity is characteristic of erysipelas. It does not remain in one place longer than three or four days, but is then apt to attack some other contiguous portion and remain there for the same length of time. When it remains in one place it is called erysipelas fixum. New surfaces are sometimes invaded before the old ones heal up. Thus the disease skips about. Whilst it is possible to transfer it from one person to another there must also be a subsoil fit to receive it.

There must be, in other words, a predisposition to the disease. Trousseau tried in vain to inoculate himself and two of his students, whilst Willan and others made the same experiment and succeeded.

A peculiarity of the disease is its freakish attacks. In hospitals or barracks it may skip certain beds and attack an individual perhaps in the other end of the room. In the Middlesex Hospital it was observed that it attacked always two adjoining beds; the cause was looked for and finally found in a defective sewerage pipe which had its exit just at this place. The pipe was repaired and the erysipelas disappeared. After a year the same observance of erysipelas was made as before, the pipes were re-examined and a defect also found again. It was repaired with the same result as before. In the amphitheatre of a small hospital in Germany it was found that all patients who had been operated upon in the amphitheatre contracted erysipelas, whilst those operated upon in their wards escaped. The cause was found in the contents of the pillows of the operating table which had been saturated by the blood of some erysipelatous patient. The proof was demonstrated by the fact that the disease was inoculated into animals from the solution in which the pillows were soaked. We know it, therefore, to be a specific disease and not to depend upon ordinary putrefactive changes as was formerly supposed. We know erysipelas to be due to bacteria. Erysipelas received its name from its red color. Because it runs over the surface it is called by the Germans *Rothlauf*, by the English *St. Anthony's Fire*, from an old saint, St. Anthony, who was sore-troubled by the lusts of the flesh which flush the skin. St. Anthony used to throw himself in a bank of snow to cure his red surface, and cold is still regarded by some clinicians as a valuable adjunct in treatment.

Another peculiarity is the fact that it spreads in a peculiar way, following the rhombic meshes of the skin. Erysipelas extends in the direction of the long axis of the skin when a round hole is punched in it. When it attacks the face it does not pass down to the chin and neck, but on the sides of the face involving the ears, and over the scalp to the occiput and neck. It also skips any part of the skin which adheres closely to subjacent bone, as at the occiput, or the spine of the leg. It is apt to attack loose structures by preference, which is explained by the arrangement of the connective tissue fibres. But no matter what part of the body is attacked it always shows a distinct line of demarcation. This distinguishes erysipelas from erythema. Erythema is often caused by indigestion, as by eating of certain shell-fish. It is also seen in the first stages of scarlatina and small-pox. It is, however, hardly ever (in its simple form) attended by fever and never shows the line of demarcation. Nodosities may appear as in the form called erythema nodosum, but these nodosities are always near the joint, are symmetrically disposed on both limbs, and are nearly always on the anterior face of the joints.

Some people have an habitual tendency to erysipelas, especially those of a scrofulous habit, but the majority of such cases, said to be often attacked by erysipelas, are really subjects of erythema. Erysipelas often becomes very severe, both in extent and character of the inflammation. It often closes the eyelids and distorts the face beyond recognition, and sometimes an exudation of pus occurs. I have seen the scalp lifted up by a layer of pus. Occasionally the disease takes the phlegmonous form when deep-seated, or extensive abscesses, furuncles or carbuncles are formed. A true carbuncle is a local affection; but carbuncular affections are found in connection with erysipelas. Both erysipelas and carbuncle occur in debilitated subjects, but whilst carbuncle is fixed to a particular region, erysipelas is characterized by its fugacity. Erysipelas is frequently ushered in by chilly sensations, and a rise of temperature, as much as 103 to 105 degrees on the first day of the disease. After the chill the redness sets in, which is, however, not always very marked in color; sometimes it is very light, so that Pirogoff spoke of a white erysipelas, *erysipelas album*, in which the skin inflamed and glistening is not marked by redness, but, as its name implies, is pallid. Ordinary erysipelas is a trivial affection, and, as said before, never lasts longer than three or four days in one place. It, however, gradually attacks other portions of the body, and its average duration is about two weeks. It is comfort to the patient to know this, for although it may be migratory in character it affords the patient some relief to have the disease shifted from one place to another.

No disinfectants will do any good in preventing erysipelas or septicemia, and if it makes its appearance in a hospital, the only prophylaxis is to close it up, or better, if severe, to burn down the institution. The true prophylaxis of this disease then requires us in the first place to abstain from treating such cases when there is any danger of infecting others. Surgeons should therefore never pass from erysipelatous cases to cases of wounds, or at the time undertake other operations. This precaution is not always possible, but surgeons should at least thoroughly disinfect their instruments, by heating them in a Bunsen's burner, or by placing them in scalding water previous to operation.

Erysipelas is at first a local and then later a general disease. Metastatic erysipelas is sometimes spoken of; it often takes the suppurative form, and is then particularly liable to attack the serous membranes as the peritoneum or the pleura in puerperal fever, the joints in rheumatism, or the kidneys in parenchymatous nephritis. These complications are all very dangerous. Facial erysipelas is liable to cause meningitis when the prognosis is very gloomy. You must, however, be careful not to infer that because a patient is delirious he has meningitis, for every high fever may lead to delirium, especially in children and aged people, but must look for other symptoms, as photophobia, twitchings or convulsions, hyperesthesia, etc.

The time of life most liable to erysipelas is between the ages of twenty and forty. It is rarer in children, but may spring up from vaccination. Thus in Boston it followed vaccination in 1854 so extensively, that this process had to be stopped for a while. Fortunately it did not occur during an epidemic of small-pox.

